# Effect of Chemical Polishing on the Formation of TiO_2_ Nanotube Arrays Using Ti Mesh as a Raw Material

**DOI:** 10.3390/nano14231893

**Published:** 2024-11-26

**Authors:** Wanshun Li, Shiqiu Zhang, Fei Li

**Affiliations:** 1 College of Chemistry, Chemical Engineering and Materials Science, Shandong Normal University, Jinan 250014, China; 202210100633@stu.sdnu.edu.cn; 2 School of Environment and Resource, Southwest University of Science and Technology, Mianyang 621010, China

**Keywords:** TiO_2_NTAs, anodic oxidation, Ti mesh, mechanism, photoelectrocatalysis

## Abstract

As a unique form of TiO_2_, TiO_2_ nanotube arrays (TiO_2_NTAs) have been widely used. TiO_2_NTAs are usually prepared by Ti foil, with little research reporting its preparation by Ti mesh. In this paper, TiO_2_NTAs are prepared on a Ti mesh surface via an anodic oxidation method in the F-containing electrolyte. The optimal parameters for the synthesis of TiO_2_NTAs are as follows: the solvent is ethylene glycol and water; the electrolyte is NH_4_F (0.175 mol/L); the voltage is 20 V; and the anodic oxidation time is 40 min without chemical polishing. However, there is a strange phenomenon where the nanotube arrays grow only at the intersection of Ti wires, which may be caused by chemical polishing, and the other areas, where TiO_2_NTAs cannot be observed on the surface of Ti mesh, are covered by a dense TiO_2_ film. New impurities (the hydrate of TiO_2_ or other products) introduced by chemical polishing and attaching to the surface of the Ti mesh reduce the current of anodic oxidation and further inhibit the growth of TiO_2_ nanotubes. Hence, under laboratory conditions, for commercially well-preserved Ti mesh, there is no necessity for chemical polishing. The formation of TiO_2_NTAs includes growth and crystallization processes. For the growth process, F^−^ ions corrode the dense TiO_2_ film on the surface of Ti mesh to form soluble complexes ([TiF_6_]^2−^), and the tiny pores remain on the surface of Ti mesh. Given the basic photoelectrochemical measurements, TiO_2_NTAs without chemical polishing have better properties.

## 1. Introduction

With the advancement of industrialization and urbanization, global warming is becoming an extremely serious issue [1,2] and CO_2_ is the biggest contributor [3]. In addition to reducing CO_2_ emissions, enhancing CO_2_ reduction into intermediate chemicals or fuels (such as HCOOH, CH_3_OH, C_2_H_5_OH, etc.) via renewable energy [4] (such as wind, electricity, solar, nuclear, etc.) is a better strategy to alleviate global warming [5,6].

The photoelectrocatalysis reduction of CO_2_ is a significant approach to transfer CO_2_ into target substances [7,8]. Nowadays, the primary representative semiconductor materials are titanium dioxide (TiO_2_), zinc oxide (ZnO), metal–organic frameworks (MOFs), graphitic carbon nitrides (g-C_3_N_4_), zeolites, etc. [9,10]. Among them, TiO_2_, as an excellent N-type semiconductor, is widely applied in photoelectrocatalysis [11,12,13,14]. Some examples include the following: TiO_2_ nanoparticles, which have strong phase stability in different environments according to thermodynamic models [15,16]; one-dimensional TiO_2_ nanowires, which can promote electron transfer along the direction of nanowires with a short carrier diffusion distance, thereby inhibiting the recombination efficiency of photogenerated e^−^ and h^+^ [17,18]; TiO_2_ nanorods, which have a high aspect ratio and promotes electron survival [19]; and TiO_2_ microspheres, which have a better light absorption capacity and high durability [20]. In recent years, TiO_2_ nanotube arrays (TiO_2_NTAs), as a new form of TiO_2_, has been extensively studied [9,21,22,23]. It has a high recovery rate and the characteristics of neat arrangement, an ordered porous formation and large specific surface area because of its long tubular and hollow structure [24]. Anodic oxidation is the most attractive method [25]. The anodic oxidation method uses titanium (Ti) as a source of anode and generates TiO_2_NTAs by applying DC voltage. In this process, the electrolyte is usually an organic solvent, such as ethylene glycol (EG) or glycerin (GI), with fluoride ions, such as ammonium fluoride (NH_4_F) [26]. The crystalline form (rutile or anatase) of TiO_2_ is generally formed in the calcination stage [27]. The length, diameter and wall thickness of the TiO_2_ nanotubes can be adjusted by changing the applied voltage, oxidation time, temperature, pH, concentration of the fluorine (F)-containing electrolyte, water content in the solvent and so on [25]. In the recent studies and application of TiO_2_NTAs, most of them use Ti foils (plates) as their Ti source via the anodic oxidation technique [28,29,30,31]. Cardoso et al. deposited ZIF-8 on the surface of TiO_2_NTAs based on Ti plates to enhance its adsorption of CO_2_ and photocatalytic reduction of CO_2_ to methanol and ethanol [32]. Ag-TiO_2_NTAs/Ti foils prepared by R. Piao et al. with electrodeposition has high visible light absorption activity and high H_2_ generation performance at 1 V [33]. LiFu et al. constructed BiVO_4_ heterojunctions and deposited N on TiO_2_NTAs/Ti foils to promote water splitting and charge transfer [34]. Different from the plate substrate of Ti foils, Ti mesh is formed by many Ti wires overlapping each other, which can provide a larger specific surface area at the same light intensity [35]. The nanotubes show radial growth on the surface of the Ti mesh, forming a three-dimensional (3D) structure, which can not only provide more reactive sites, but also be favorable for light scattering and have a greater utilization of solar energy. The 3D radially grown TiO_2_ nanotubes can collect incident light from any direction, reducing the dependence on the angle of incident light [36]. Under the same demand, less raw materials (Ti metal) are needed, which can save costs and obtain greater economic benefits. For the pretreatment process of raw material, sandblasting, mechanical polishing and organic matter ultrasound, especially pickling (chemical polishing), are effective ways to clean and remove surface impurities. However, for Ti mesh, some scholars choose to carry out chemical polishing [35,37,38], that is, chemical corrosion through aqueous solutions of HF and HNO_3_ (polishing solution), while some scholars choose not to carry out polishing [32,39]. However, the effect of chemical polishing on the growth of TiO_2_NTAs is not clear and still needs more research to explore.

In this study, TiO_2_NTAs were prepared by anodizing, based on Ti mesh, through a polishing time variable experiment. Furthermore, we found that after immersing the Ti mesh in a polishing solution, TiO_2_NTAs could not be correctly prepared and there were nanotube structures only in small areas. Chemical polishing was found to reduce the current value during the anodic oxidation, which is not conducive to the growth of TiO_2_ nanotubes. Finally, basic photoelectrochemical measurements were carried out and the synthesis mechanism of TiO_2_NTAs is explained herein.

## 2. Materials and Methods

### 2.1. Reagents

The dimensions of pure Ti mesh are 0.25 mm × 10 mm × 25 mm. Anhydrous ethanol (C_2_H_6_O, AR: ≥99.7%) was purchased from Tianjin FUYU Chemical Co., Ltd. (Tianjin, China). Acetone (C_3_H_6_O, AR: ≥99.5%) was obtained from Yantai Yuandong Fine Chemicals Co., Ltd. (Yantai, China). Ethylene glycol (EG, C_2_H_6_O_2_, AR: ≥99.5%, CAS:107-21-1), ammonium fluoride (NH_4_F, AR: ≥96.0%, CAS:12125-01-8), sodium fluoride (NaF, AR: ≥98%, CAS:7681-49-4), potassium fluoride dihydrate (KF·2H_2_O, AR: ≥99.0%, CAS:13455-21-5), nitric acid (HNO_3_, AR: 65.0~68.0%, CAS:7697-37-2) and hydrofluoric acid (HF, AR: ≥40.0%, CAS:7664-39-3) were purchased from Sinopharm Chemical Reagent Co., Ltd. (Shanghai, China). Sodium sulfate anhydrous (Na_2_SO_4_, AR, 99%, CAS: 7757-82-6) was purchased from Shanghai Macklin Biochemical Co., Ltd. (Shanghai, China). Finally, the deionized water (H_2_O), which was used in all experiments, was produced by Hangzhou Wahaha Group Co., Ltd. (Hangzhou, China).

### 2.2. TiO_2_NTA Preparation

**Figure 1:**
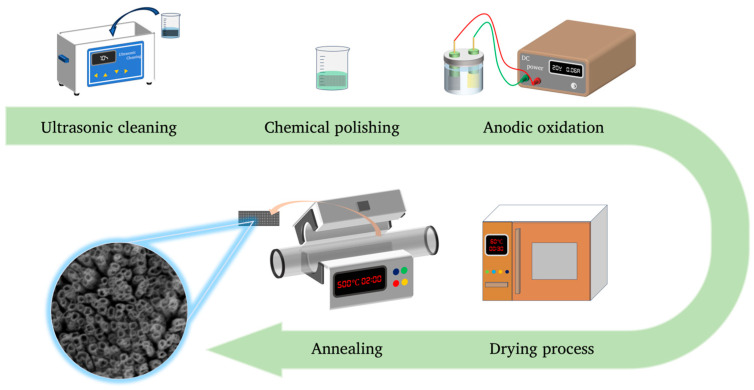
Flow chart of TiO_2_NTA preparation.

The Ti mesh was firstly washed with acetone and deionized water for 10 min, respectively, with ultrasound-assisted cleaning. Then, the Ti mesh was immersed in the polishing solution for chemical polishing and thrown into deionized water to undergo the quenching reaction. The polishing solution was prepared by mixing deionized water, HF and HNO_3_ (*V*:*V*:*V* = 2:1:1). Titanium dioxide nanotube arrays (TiO_2_NTAs) were prepared by anodizing. The electrolyte was prepared using EG and H_2_O (*V*:*V* = 2:1), and NH_4_F (KF or NaF) was the solute whose concentration was 0.175 mol/L. The Ti mesh was the anode and the platinum mesh was the cathode. The distance between the electrode surfaces was 3 cm. The set voltages were 10 V, 20 V, 30 V and 40 V with the DC power supply (IT6823), which was purchased from ITCH Electronic Co., Ltd. (Nanjing, China). The oxidation times were to 20 min, 40 min, 60 min, 80 min, 120 min and 180 min. After anodizing, the Ti mesh was washed with deionized water 3 times to remove the solute and solvent on the surface of the Ti mesh. Subsequently, the Ti mesh was dried in an oven for 30 min at 60 °C. Then, the Ti mesh was calcined at 500 °C (also known as the annealing treatment) in a tube furnace for 2 h with the quiescent air at a heating rate of 3 °C/min (The TiO_2_NTA preparation process detailed in Figure 1). After annealing, natural cooling was performed and the cooling time varied with the ambient temperature. According to the above experimental parameters, we prepared three groups of samples by changing other treatment methods (electrolyte, anodizing time and chemical polishing time) for Ti mesh. Finally, all samples were put in 5 mL test tubes for data measurements.

### 2.3. Variable Setting

***Electrolyte:*** In addition to NH_4_F, NaF and KF (0.175 mol/L) were selected as electrolytes. The voltages were 10 V, 20 V, 30 V and 40 V. The samples are denoted as A-NT-10, A-NT-20, A-NT-30, A-NT-40, K-NT-10, K-NT-20, K-NT-30 and K-NT-40 (A is for NaF and K is for KF). The anodic oxidation time was 180 min and the electrolyte was still composed of EG and water (*V*:*V* = 2:1). The annealing operation was as the procedure described above. ***Anodizing time:*** The reaction times were 20, 40, 60, 80, 120 and 180 min with a voltage of 20 V at room temperature, and the electrolyte was NH_4_F (0.175 mol/L). The samples are sequentially written as NT-20, NT-40, NT-60, NT-80, NT-120 and NT-180. ***Chemical polishing time:*** The polishing times were changed to 0, 4, 6, 8, 10, 12, 16 and 20 s to prepare TiO_2_NTAs with a voltage of 20 V and an anodizing time of 40 min. A polishing time of 0 s indicates that chemical polishing was not performed. The samples are sequentially written as P0-NT-40, P4-NT-40, P6-NT-40, P8-NT-40, P10-NT-40, P12-NT-40, P16-NT-40 and P20-NT-40. ***Blank control samples*:** A Ti mesh (named as P0-TM) was only cleaned using acetone and deionized water, not polished or anodized; another (named as P10-TM) was cleaned and chemically polished for 10 s, without anodizing.

### 2.4. Characterizations

The surface morphology and structure of the samples were observed by scanning electron microscopy (SEM, Regulus 8010, Hitachi High-Tech Corporation, Tokyo, Japan). The X-ray diffractograms of the sample are obtained by an X-ray diffractometer (XRD, SmartLabSE, Rigaku Corporation, Tokyo, Japan) to analyze the crystal structure. Angles of 2*θ* ranged from 10° to 80° degrees with a scan rate of 20°/min and steps of 0.01°. Sample elements and their valence information were obtained by an X-ray photoelectron spectroscopy (XPS, Escalab 250Xi, Thermo Fisher Scientific, Waltham, MA, USA) instrument with Al-Kα as a radiation source and energy dispersive spectrometer (EDS). TiO_2_NTAs’ photoelectrochemical property was measured using an electrochemical workstation (CHI660E, CH Instruments Inc., Shanghai, China) purchased from Shanghai Chuang Hua Instrument Co., Ltd. (Shanghai, China). TiO_2_NTAs were used as the working electrode, platinum mesh as the counter electrode, and Ag/AgCl as the reference electrode. The light source was a 300 W xenon lamp and the electrolyte was 0.5 mol/L Na_2_SO_4_.

### 2.5. Correlation Formula

#### 2.5.1. Applied Bias Photon-to-Current Efficiency (ABPE)


$$ABPE=\frac{\left(1.23V-\left|V_{a}\right|\right)\times j_{p}}{P_{light}}\times 100\%$$

V_a_: Applied bias voltage/V vs. RHE;

j_p_: Photocurrent density/mA·cm^−2^;

P_light_: Optical power density/mW·cm^−2^.

## 3. Results and Discussion

### 3.1. Morphology Analysis

In this section, we focus on whether TiO_2_NTAs have been successfully generated on the surface of a Ti mesh via the anodic oxidation method. Through SEM scanning images, we can have an extremely intuitive and clear understanding of the condition of the surface of the Ti mesh.

#### 3.1.1. Effect of Electrolyte

**Figure 2:**
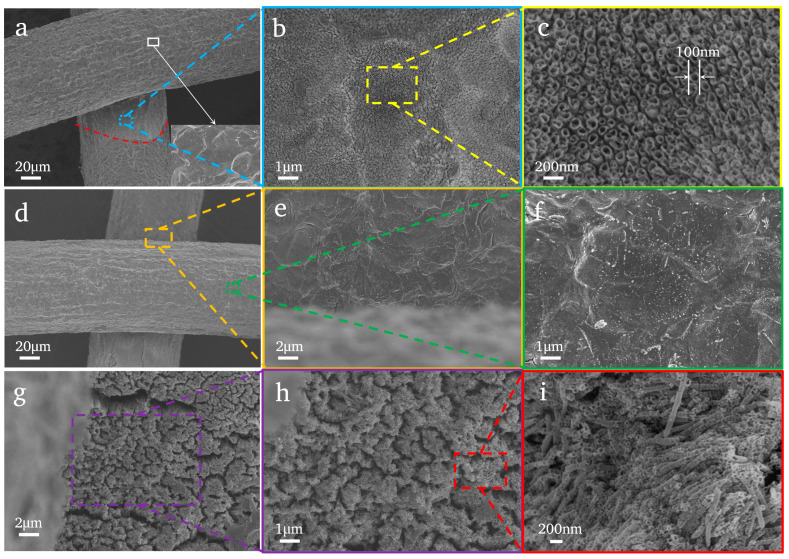
SEM images of the TiO_2_NTAs in variable electrolytes: (**a**–**c**) K-NT-20; (**d**–**f**) A-NT-20; (**g**–**i**) N-NT-20.

Figure 2 shows the SEM image results of the samples in different electrolytes. We selected a series of electrolytes (KF, NaF and NH_4_F) to anodize the Ti mesh. The images indicate the following: (i) When the electrolyte is KF (Figure 2a–c), there are only TiO_2_ nanotubes on the intersection of two Ti wires, and the diameter of these nanotubes is approximately 100 nm in average (Figure 2c). In addition, the difference between them is that the morphology of the nanotubes prepared by KF is malformed (Figure 2c). (ii) When the electrolyte is NaF, there is no nanotube structure, not only on the surface of the Ti mesh, but also in the intersection, by just a few dents (Figure 2d–f). (iii) When NH4F is the electrolyte, a large number of nanotubes (Figure 2g–h) appear on the surface of the Ti mesh. However, the nanotubes are disorderly (Figure 2i), which may be due to the excessively long anodizing time leading to a nanotube length that is too long and a broken structure. Then, we adjusted the anodizing time to explore the appropriate time.

#### 3.1.2. Effect of Anodizing Time

**Figure 3:**
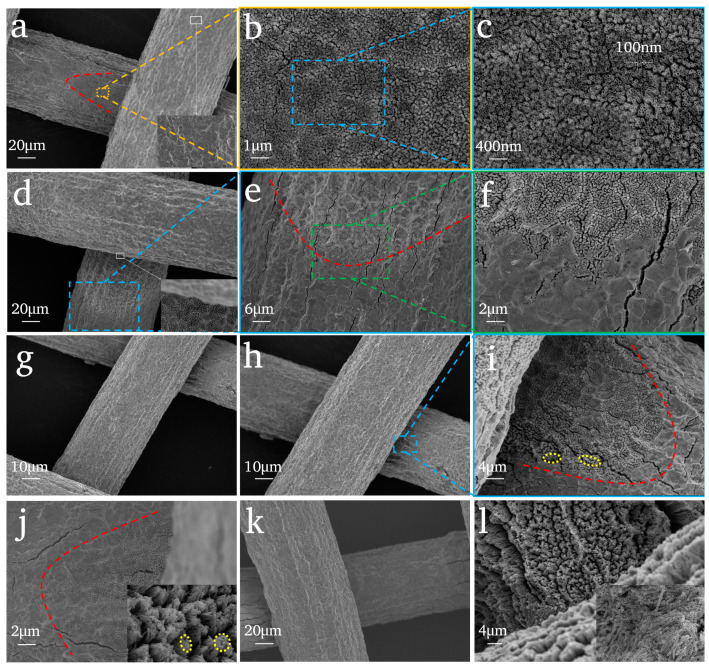
SEM images of TiO_2_NTAs prepared by variable anodizing time: (**a**–**c**) 20 min; (**d**–**f**) 40 min; (**g**–**i**) 60 min; (**j**) 80 min; (**k**) 120 min; (**l**) 180 min. Conditions: Ti mesh substrate; chemical polishing and anodizing in 20 V with 0.175 mol/L NH_4_F.

Figure 3 shows the microstructure of TiO_2_NTAs caused by anodizing time. It indicates the following: (i) There is a very strange phenomenon for all samples as the TiO_2_ nanotube structure only appears in the intersection of two Ti wires and the phenomenon is the same as that on the samples where KF is selected as the electrolyte (Figure 2a). (ii) As shown in Figure 3a, there are only some shallow pits on the surface of the Ti mesh except at the intersection of the two Ti wires. In areas where there are TiO_2_NTAs, the diameter of the TiO_2_ nanotubes is approximately 100 nm (Figure 3c). When the anodizing time is 20 min, the NTA structures arrange themselves densely and neatly grow (Figure 3b,c). (iii) With the extension of anodizing time, the nanotube length gradually increases. At the time of 60 min, the TiO_2_NTAs are no longer neatly arranged, and they start to gather. When the time is 80 min, this phenomenon is extremely significant (the yellow dotted line area has been marked in Figure 3i,j). (iv) When the anodizing time is 180 min, the TiO_2_NTAs break and are disorderly and unsystematic, which may be due to the long lengths of the nanotubes. (v) There is a clear dividing line (the red dotted line is marked in Figure 3a,e,i,j): one side of the dividing line is the nanotube structure, and the other side is the concave structure, extending to one side from the intersection of the Ti wires.

#### 3.1.3. Effect of Chemical Polishing Time

**Figure 4:**
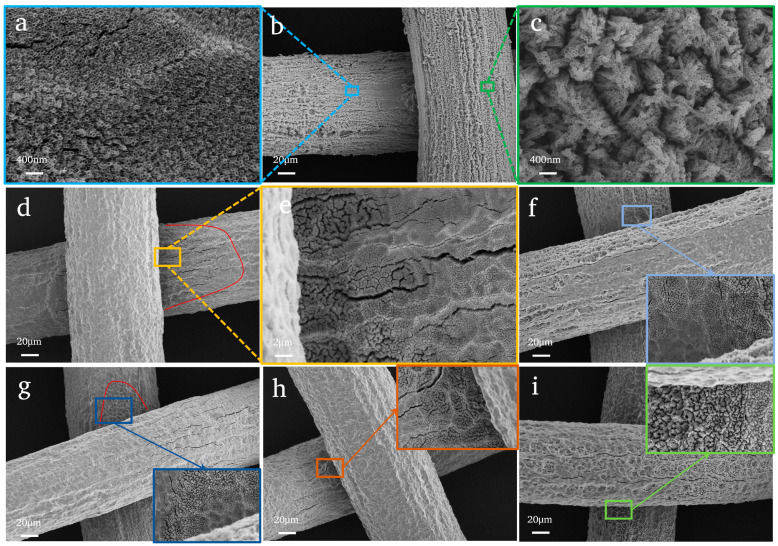
SEM images of TiO_2_NTAs prepared by variable chemical polishing time: (**a**–**c**) P0-NT; (**d**,**e**) P4-NT; (**f**) P6-NT; (**g**) P8-NT; (**h**) P10-NT; and (**i**) P12-NT. Conditions: Ti mesh substrate; anodizing time 40 min and voltage 20 V with 0.175 mol/L NH_4_F.

The SEM images below (Figure 4) are the TiO_2_NTAs prepared by variable chemical polishing time. They indicate the following: (i) When the chemical polishing time is 0 s, the above strange phenomenon does not appear, and TiO_2_NTAs appear on all areas of the surface of the Ti mesh (not only on the intersection of Ti wires) (Figure 4a–c). (ii) Once the Ti mesh is polished, no matter how long the polishing time is, the TiO_2_NTAs cannot grow well on the surface of the Ti mesh. And most areas of the surface of the Ti mesh are pits. (iii) When the polishing time is more than 16 s, the Ti mesh has been softened and is not suitable for preparing the TiO_2_NTAs, so the samples with polishing time 16 s and 20 s are not analyzed by SEM.

From the above results, it is concluded that (i) the growth of TiO_2_NTAs on the surface of a Ti mesh is not as good as that on the surface of Ti foil [40], and (ii) when the Ti mesh is polished by chemical reagents, the TiO_2_NTAs cannot grow well. The TiO_2_NTAs only exist at the intersection of Ti metal wires. This strange phenomenon should be further explored.

### 3.2. XRD Analysis

#### 3.2.1. Effect of Electrolyte

**Figure 5:**
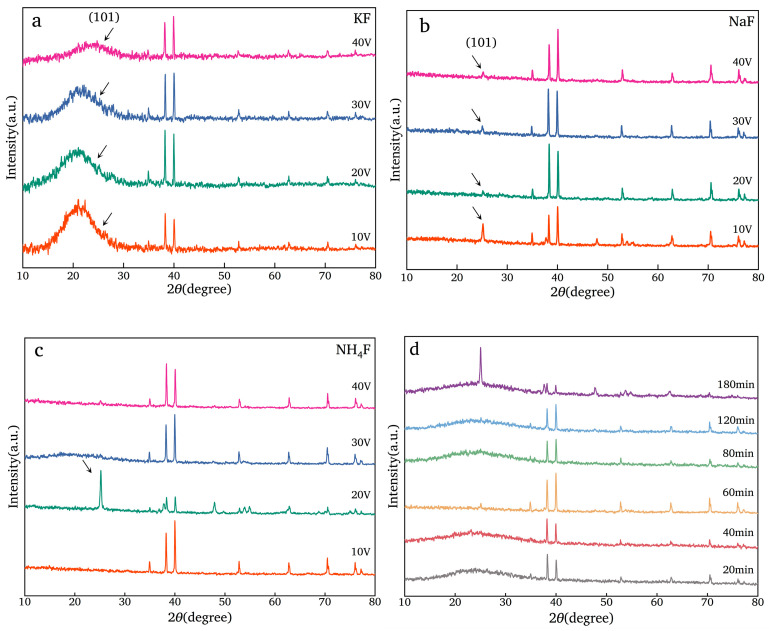

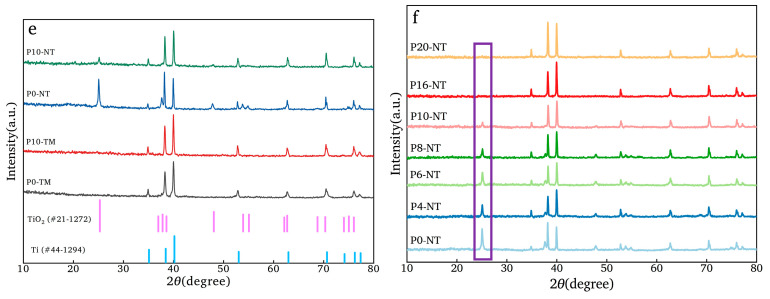
(**a**–**c**) XRD results of TiO_2_NTAs prepared by different electrolytes: (**a**) KF; (**b**) NaF; (**c**) NH_4_F. (**d**) XRD results of TiO_2_NTAs prepared by different anodizing time. (**e**) XRD results of P0-TM, P10-TM, P0-NT and P10-NT. (**f**) XRD results of TiO_2_NTAs prepared by different polishing time.

When the electrolytes are NaF or KF, the intensity of the characteristic peak of TiO_2_ near 25.28°, attributed to the (101) crystallographic plane, is almost weak (Figure 5), which may have been caused by the crystallization of the dense TiO_2_ film on the surface of the Ti mesh after annealing. When KF is selected as the electrolyte (Figure 5a), there is an obvious and broad peak near 25.28°, which indicates that the amorphous substance is produced. When the electrolyte is NH_4_F and the voltage is 20 V (Figure 5c), the intensity of the characteristic peak of TiO_2_ near 25.28°, attributed to the (101) crystallographic plane, is extremely strong compared to the other samples.

#### 3.2.2. Effect of Anodizing Time

The length of the TiO_2_NTAs and the thickness of the TiO_2_ film (detailed in the growth mechanism of TiO_2_NTAs) on the Ti mesh surface are primarily affected by the anodizing time. When the anodizing time is less than 180 min (Figure 5d), there is no obvious characteristic peak attributed to the (101) crystallographic plane. When the anodizing time is 180 min, the intensity of the (101) characteristic peak is particular, indicating that the crystallization of TiO_2_NTAs on the surface of the Ti mesh is perfect.

#### 3.2.3. Effect of Chemical Polishing Time

XRD results of four samples are presented in Figure 5e: the Ti mesh before and after chemical polishing without anodizing (P0-TM and P10-TM) and the Ti mesh before and after polishing with anodizing (P0-NT and P10-NT). If the sample is not anodized, there is no change in the crystal structure of the Ti mesh, regardless of the chemical polishing (P0-TM and P10-TM). When the Ti mesh is anodized directly without chemical polishing (Figure 5e, P0-NT), the XRD results of this sample could be matched by TiO_2_ and Ti. The diffraction peaks at the degree of 2*θ* are 25.28°, 36.95°, 37.80°, 38.58°, 48.05°, 53.89°, 55.06°, 62.12°, 62.69°, 68.76°, 70.31°, 74.03°, 75.03° and 76.02°, attributed to the (101), (103), (004), (112), (200), (105), (211), (213), (204), (116), (220), (107), (215) and (301) crystallographic planes of the anatase standard card (PDF#21-1272), respectively. The sharp shape of the diffraction peak indicates that the degree of crystallinity of TiO_2_ is perfect. When the Ti mesh is chemically polished and then anodized, the intensity of these diffraction peaks decreases (Figure 5e, P10-NT), which may be caused by the decrease in the crystallization content of TiO_2_NTAs on the surface of the Ti mesh. With the increase in polishing time (Figure 5f), the intensity of the characteristic peak of TiO_2_NTAs (2*θ* = 25.28°) gradually decreases (detailed the purple box in Figure 5f). When the polishing time is more than 16 s, the characteristic peak of TiO_2_NTAs disappears. This may be caused by the too long polishing time, generating the Ti complexing compound, which could inhibit the TiO_2_NTAs’ growth on the surface of the Ti mesh.

Combined with the results of morphology analysis and XRD analysis on all the TiO_2_NTAs samples, we can determine that the appearance of TiO_2_NTAs only at the intersection of Ti wires is caused by chemical polishing, and the details of TiO_2_NTA preparation using Ti mesh as a raw material is as follows: the Ti mesh itself is not chemically polished and NH_4_F is used as the electrolyte; the anodizing time is 40 min and the oxidation voltage is 20 V, ensuring that the TiO_2_NTAs have a high crystallinity degree and perfect morphology.

### 3.3. Chemical Component Analysis

#### 3.3.1. XPS Analysis

**Figure 6:**
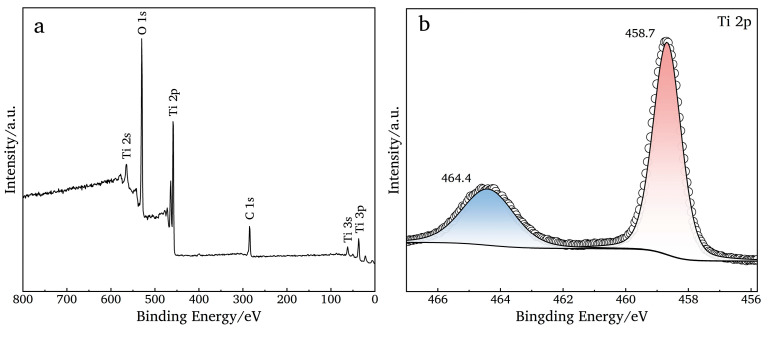

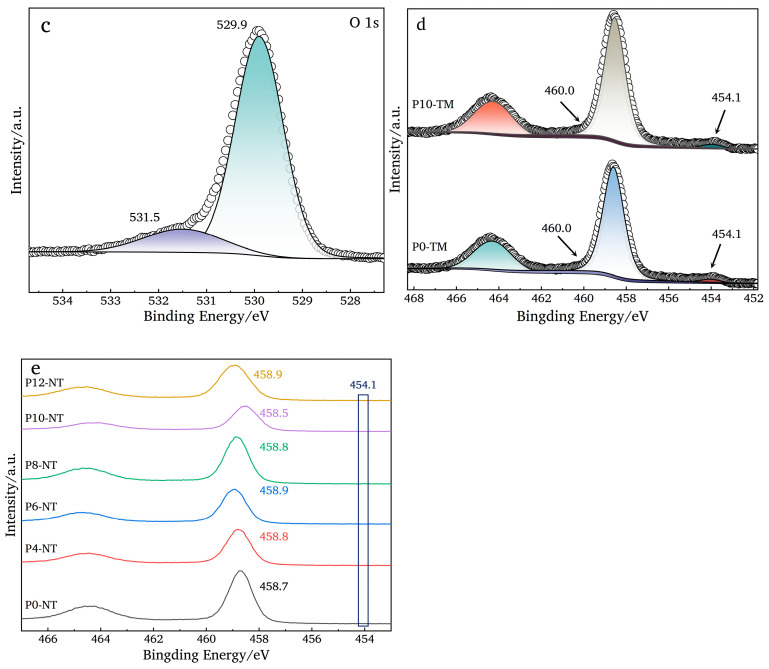
(**a**–**c**) XPS energy spectra of sample P10-NT ((**a**) full spectrum; (**b**) Ti 2p; and (**c**) O 1s); (**d**) Ti 2p fine spectrum of P0-TM and P10-TM; (**e**) Ti 2p fine spectrum of TiO2NTAs in different chemical polishing times.

The detection depth of SEM is in the nanoscale. For the full spectrum (Figure 6a), there are Ti and O elements on the surface of the Ti mesh after polishing and anodizing. For the Ti 2p spectrum (Figure 6b), the binding energies at 458.7 eV (Ti 2p_3/2_) and 464.4 eV (Ti 2p_1/2_) are attributed to Ti^4+^ [41,42], respectively. For the O 1s spectrum (Figure 6c), the binding energies at 529.9 eV and 531.5 eV are attributed to the Ti-O bond and O-H bond [24,41]. It indicates that TiO_2_ is successfully prepared on the surface of the Ti mesh. When only cleaning the Ti mesh (Figure 6d, P0-TM) or cleaning and polishing it for 10 s (Figure 6d, P10-TM) without anodizing, the binding energies at 454.1 eV (Ti 2p_3/2_) and 460.0 eV (Ti 2p_1/2_) are attributed to the Ti mesh, and the production of TiO_2_ is caused by air oxidation and the chemical polishing of the Ti mesh. When the Ti mesh is anodized, the binding energy (454.1 eV) of the Ti mesh is not detected (Figure 6e), which may be caused by the thickening of the TiO_2_ film layer after anodizing, and the binding energy (Ti 2p_3/2_) of TiO_2_ has a small degree of deviation.

#### 3.3.2. EDS Analysis

**Figure 7:**
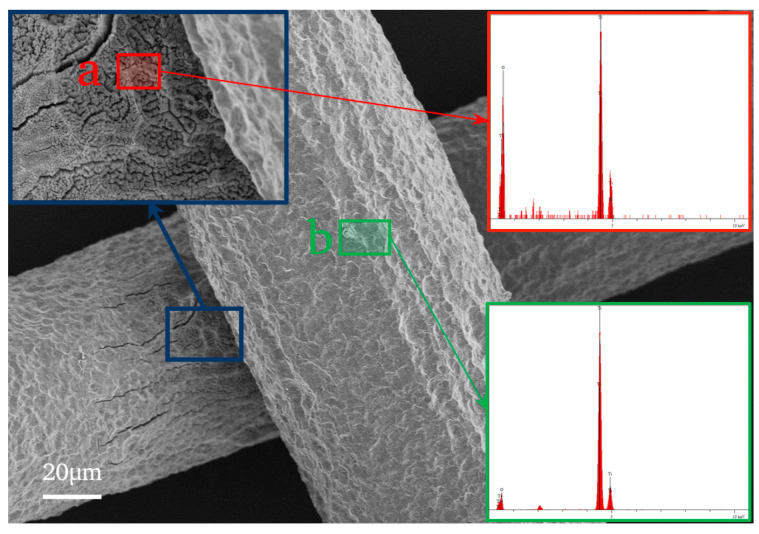
The areas of EDS test (the image of P10-NT).

A point-to-point analysis of the chemical components on the surface of the Ti mesh (Figure 7, P10-NT) was performed using SEM equipped with EDS, whose detection depth is at a micron scale. The red area is the TiO_2_NTA area and the green area is the pit area on the surface of the Ti mesh. According to the EDS results (detailed in the Table 1) and the above XPS results, the red area is the TiO_2_, and the green area is just a bit of the TiO_2_. Ti metal is detected as EDS punched through due to a thinner layer of TiO_2_, thus the content of Ti (95.228%) is bigger than that of O (4.772%). Overall, the area where the TiO_2_NTAs do not appear is a thin TiO_2_ film layer, and anodizing thickens the TiO_2_ film.

### 3.4. Explanation of Strange Phenomena

**Figure 8:**
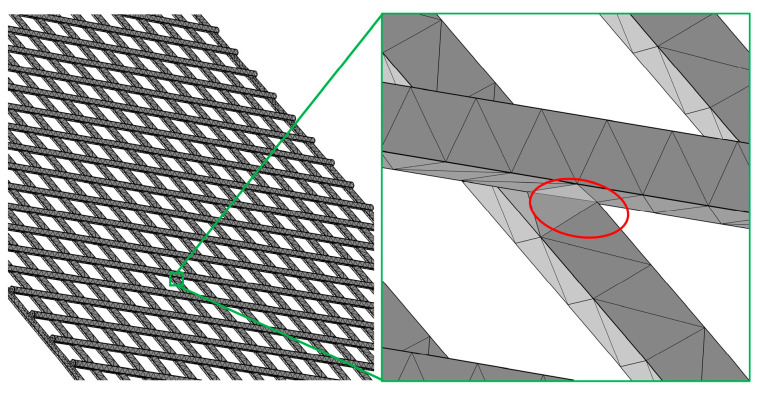
Ti mesh structure diagram.

Figure 8 shows the Ti mesh structure, and the red area (the intersection of Ti wires) is where the TiO_2_ nanotubes appear after polishing. The anodic oxidation current value of the Ti mesh after chemical polishing is much smaller than that of the unpolished (only cleaned by acetone and deionized water) directly anodizing Ti mesh, which is caused by chemical polishing that thickens the TiO_2_ film on the surface of the Ti mesh. When chemical polishing is carried out, the intersection of Ti wires has less contact with the chemical polishing solution, the polishing time is slightly shorter than that of the normal Ti mesh surface (not the intersection of Ti wires), and the thickness of the TiO_2_ film is smaller. In the anodizing process, the voltage at the intersection of Ti wires is slightly bigger than that in other areas, and it is more easily eroded by F^−^ ions. These are the reasons why the TiO_2_ nanotubes only appear on the intersection of Ti wires after chemical polishing.

### 3.5. Proposed Synthesis Mechanism

#### 3.5.1. Growth Mechanism

**Figure 9:**
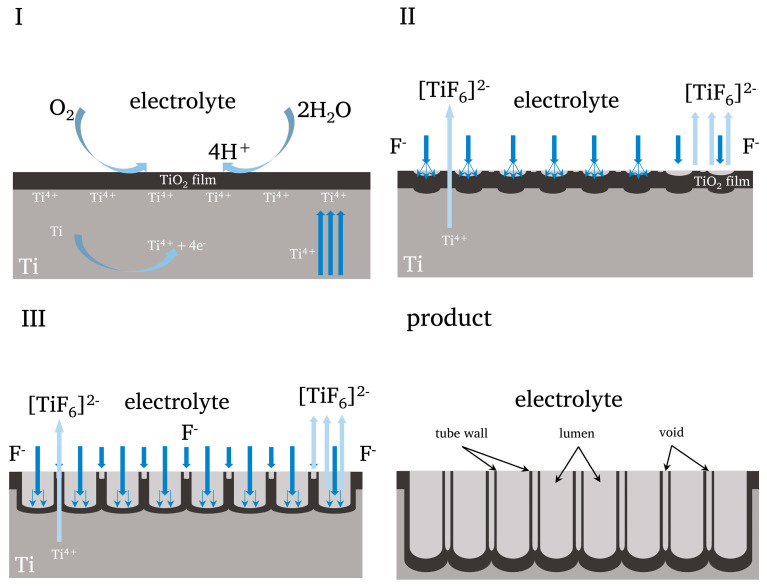
Growth mechanism picture of TiO_2_NTAs.

Based on above results and published research, we proposed a possible synthesis and growth mechanism of TiO_2_NTAs in the perspective of an applied electric field [9,43,44,45]. The formation of TiO_2_NTAs is a complex process involving physics, chemistry, and especially electrochemistry. The background of this mechanism is that the Ti mesh is connected to the anode of the cell, and the applied voltage (definite value) is added. The electrolyte is a mixture of organic solvent, water and F^−^ ions (usually NH_4_F). According to the field-assisted dissolution mechanism [46,47], the growth process of nanotubes can be roughly divided into three stages (Figure 9).

**Stage 0** is the chemical polishing process. The main purpose of chemical polishing is to remove impurities and oxide layers (TiO_2_) on the surface of the Ti mesh and enhance surface smoothness, so that the Ti metal can directly react in the anodizing step. In fact, in the case of a well-preserved commercial Ti mesh for laboratory consumables, the Ti mesh is not easily oxidized by air, so the surface of the Ti mesh will not have an oxide layer that is too thick (in the XPS results, the unpolished Ti mesh (P0-TM) can detect the binding energy of the Ti metal, indicating that the oxide layer is very thin). However, the process of pickling with a polishing solution is a very rapid process, and the polishing time is difficult to control. When the polishing time is short, the oxide layer on the surface of the Ti mesh can be removed; if the polishing time is longer, the polishing liquid will further react with the Ti metal (Equations (1)–(3)). Under laboratory conditions, the thin oxide layer boosts the probability of the latter reaction, and the hydrate of TiO_2_ is produced (Equations (2) and (3)). Simultaneously, the pickling product (also named “new impurities”, i.e., the hydrate of TiO_2_ or other products) produced by polishing will not completely dissolve in the polishing solution, and a part of it will be attached to the surface of the Ti mesh, which is contrary to the purpose of polishing. These new impurities will reduce the current value and inhibit the growth of the TiO_2_ nanotubes during anodic oxidation. The numerical performance during the experiment is that, at the beginning of anodizing, the initial current value of the Ti mesh without chemical polishing is 0.16 A, while the Ti mesh with chemical polishing is only 0.06 A, which is one of the reasons that after polishing, the TiO_2_ nanotubes only appear at the intersection of the Ti wires.

2Ti + 6HF → 2TiF_3_ + 3H_2_ ↑ (1)

3Ti + 4HNO_3_ + 4H_2_O → 3TiO_2_·2H_2_O (H_4_TiO_4_) + 4NO ↑ (2)

3Ti + 4HNO_3_ + H_2_O → 3TiO_2_·H_2_O (H_2_TiO_3_) + 4NO ↑ (3)

3Ti + 4HNO_3_ + 12HF → 3TiF_4_ + 8H_2_O + 4NO ↑ (4)

**Stage Ⅰ** is the electric field-assisted oxidation. Firstly, under the effect of an applied electrodynamic potential, the Ti mesh is oxidized and changes to form Ti ions (Ti^4+^) in the electrolyte (Equation (4)). At the same time, there is the presence of dissolved oxygen or water in the electrolyte, which could combine with Ti^4+^ to form TiO_2_ (Equations (6) and (7)), that is a dense and strong TiO_2_ film (also named as the barrier layer) on the surface of the Ti mesh. The formed TiO_2_ film is located on the Ti metal–oxide interface, rather than the oxide–electrolyte interface. The TiO_2_ film prevents further anodizing reactions, with the thickness of the film increasing and leading to the decrease in current value and oxidation rate [48]. In addition, **Stage Ⅰ** is a very short process. It should be noted that this is the only part of Ti^4+^ in this stage that would participate in the other reaction (Equation (9)). Furthermore, the fluoride ions (F^−^) do not participate in the anodizing reaction [43]. The correlation reaction equations are as follows:

Anodizing reaction:

Ti → Ti^4+^ + 4e^−^ (5)

Film-forming reaction:

Ti^4+^ + 2H_2_O → TiO_2_ + 4H^+^ (6)

Ti + O_2_ → TiO_2_ (7)

**Stage Ⅱ** is the electric field-assisted dissolution stage. At this stage, F^−^ ions and TiO_2_ form the soluble complexes ([TiF_6_]^2−^), and part of the Ti escapes from the oxide film (Equation (8)), which occurs at the interface between the electrolyte and the TiO_2_ film. In particular, the concave or thin areas (the electric field force larger than the concave edge) on the surface of the TiO_2_ film are more possibly eroded by F^−^ ions [49], which leads to the dissolution reaction. Due to the presence of the applied voltage, the strength of the Ti-O bond in the TiO_2_ decreases, facilitating [TiF_6_]^2−^ formation. The corrosion and dissolution of the oxide film by F^−^ generates many tiny voids on the oxide surface. With the increase in and growth of tiny pores, the reaction area also expands and deepens. Gradually, the process enters **Stage Ⅲ** and the current has a small increase.

Dissolution reaction:

TiO_2_ + 6F^−^ + 4H^+^ → [TiF_6_]^2−^ + 2H_2_O (8)

Ti^4+^ + 6F^−^ → [TiF_6_]^2−^ (9)

**Stage Ⅲ** is the electric field-assisted growth stage. There is no clear boundary between **Stages Ⅱ** and **Ⅲ,** and the anodization and dissolution reactions are carried out simultaneously. Initially, the dissolution reaction rate is higher than the anodization rate, which corresponds to the increase in the current in the **Stage Ⅱ**. In the center of the holes, the deepness of the oxide film is less than that of the hole’s edge. The large electric field force leads to the weak Ti-O bond, which is easier to be eroded by F^−^ ions to form [TiF_6_]^2−^. According to the equifield strength mechanism [50], the diameter of micropores will gradually increase after the initial stage of formation. After the formation of the nanotube structure, the diameter of these tubes does not increase [51]. There is a directional migration of cations to the cathode, and anions to the anode, due to the action of the electric field in the electrolytic cell, that is, Ti^4+^ tends to move away from the Ti mesh to form TiO_2_ or [TiF_6_]^2−^ and F^−^ tends to move towards to the Ti mesh and continue to erode the TiO_2_ film. This is also the driving force behind the formation of the extra nanotubes.

With the reaction continuing, the whole system is in an equilibrium state, the anodization reaction rate and the dissolution reaction rate are equal [52], and the current value is stable. Then, there is the steady growth of the TiO_2_ nanotubes to finally form the TiO_2_ nanotube arrays.

#### 3.5.2. Crystallization Process

The anodized TiO_2_ is amorphous. The photocurrent of the crystallized TiO_2_ nanotubes can be significantly improved compared to that of the uncrystallized TiO_2_ nanotubes [53,54]. The way to crystallize TiO_2_ is to anneal it in a tube furnace [55]. The temperature is generally 400–500 °C, and the annealing time is 1–3 h [56]. After annealing, the diffraction peak of TiO_2_ increases, and simultaneously, the peak intensity of the Ti metal decreases [57]. Usually, the crystal form of TiO_2_ nanotubes after annealing is anatase, which is consistent with the experimental results. If one should want to obtain a rutile crystal, they would generally need an annealing temperature greater than 600 °C [58]. When the annealing temperature reaches 650 °C, two phases (66% anatase and 34% rutile) will appear [59]. After annealing, especially in an atmosphere with low oxygen content, TiO_2_ has a higher oxygen vacancy density, which is conducive to increasing the absorption of visible light and promoting charge transfer [60,61].

## 4. Photoelectric Chemical Property Analysis

### 4.1. Linear Sweep Voltammetry (LSV) Test

**Figure 10:**
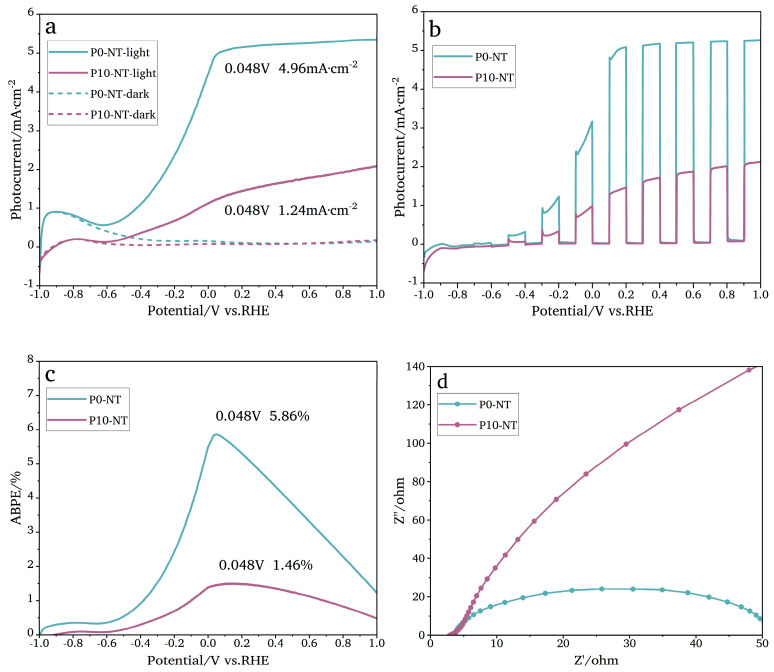
(**a**) The LSV curve of P0-NT and P10-NT in the light and dark; (**b**) photocurrent response curves of TiO_2_NTAs; (**c**) ABPE curves of TiO_2_NTAs P0-NT and P10-NT; and (**d**) the electrochemical impedance spectrum of TiO_2_NTAs P0-NT and P10-NT.

Figure 10a shows the photocurrent of TiO_2_NTAs (P0-NT and P10-NT) whether there is light or not. When there is no light, the current is low and even approaches zero. The photocurrent is much higher in the light than that in the absence of light. The P0-NT photocurrent is 4.96 mA·cm^−2^ (0.048 V), which is 4.34 times than that of P10-NT (1.24 mA·cm^−2^, 0.048 V), and keeps constant as the voltage increases. This is due to the large areas of the TiO_2_NTAs on the surface of the Ti mesh. Figure 10b shows when the light is turned on or off every 20 s. When the voltage is less than 0.2 V, small sharp peaks appear at each cycle, which may be caused by a faster recombination of e^−^ and h^+^ on the TiO_2_NTA surface or capacitive current. Figure 10c shows the ABPE curves obtained by calculating with the formula detailed in Section 2.5.1. When the applied bias voltage is 0.048 V, the ABPE of TiO_2_NTAs P0-NT is 5.86%, 4.01 times than that of P10-NT (1.46%).

### 4.2. EIS Analysis

Based on the electrochemical impedance spectroscopy (EIS) test (Figure 10d), the charge transfer ability of TiO_2_NTAs P0-NT and P10-NT is displayed. The circular arc radius of P0-NT is smaller than that of P10-NT. This indicates that the charge transfer impedance of P0-NT is low. TiO_2_NTAs prepared without chemical polishing (P0-NT) have a stronger charge transfer ability. This may also be caused by smaller areas of TiO_2_ nanotubes on the surface of the Ti mesh. To sum up, TiO_2_NTAs prepared by Ti mesh without chemical polishing have better photoelectric chemical properties, according to the LSV and EIS tests.

## 5. Conclusions

TiO_2_NTAs are prepared on a Ti mesh surface via an anodic oxidation method using different electrolytes, changing the anodizing time and the chemical polishing time. NH_4_F is an excellent electrolyte for growing uniform, well-ordered, high-quality TiO_2_ nanotubes, compared to KF and NaF. In particular, when NaF is used as an electrolyte, there is no nanotube structure produced. The anodizing time mainly affects the length of the nanotubes. (i) After a brief anodizing time (20–40 min), the TiO_2_ nanotubes are neatly arranged and densely packed; (ii) after an intermediate anodizing time (60–80 min), the arrangement of the TiO_2_ nanotubes is gradually disorganized and clustered; and (iii) after a long anodizing time (120–180 min), the TiO_2_ nanotubes are disordered and unsystematic. Chemical polishing interrupts the uniform growth of nanotubes on the surface of the Ti mesh. (i) When no chemical polishing is performed, the TiO_2_ nanotubes can grow in all areas of the Ti mesh and be tightly arranged with the intense characteristic peaks of anatase. (ii) When the chemical polishing time is moderate (4–12 s), there is the strange phenomenon that the nanotube arrays grow only at the intersection of the Ti wires, and the other areas, where TiO_2_NTAs cannot be observed on the surface of the mesh, are covered by a dense TiO_2_ film. (iii) When the chemical polishing time is too long, the Ti mesh softening is not suitable for nanotube growth, and there are no characteristic anatase peaks. Optimal anodizing time and voltage are critical for preserving uniformity and structural integrity. Moreover, the proposed formation mechanism of TiO_2_NTAs includes the growth mechanism and crystallization mechanism. The photoelectric chemical properties of TiO_2_NTAs without chemical polishing are more perfect than those with polishing for 10 s. For the chemical polishing of the Ti mesh, although the surface oxide layer can be removed, new impurities (the hydrate of TiO_2_ or other products) are introduced and attach to the surface of the Ti mesh, which does not optimize the anodic oxidation process, but inhibits the growth of the TiO_2_ nanotubes. And the post-treatment of HF and HNO_3_ in the laboratory and NO produced by the pickling process are harmful to the environment, increase costs, and do not bring certain economic benefits. This is more harm than good for commercial Ti mesh. Hence, when the Ti mesh is used as a Ti source, it can be cleaned directly with acetone and deionized water for anodizing without the necessity for chemical polishing.

In addition to this, more attention should be paid to the influence of other components of polishing solutions (different proportions of HF, HNO_3_ and water or other acids) on the preparation of TiO_2_ nanotube arrays under various condition variables (voltage, electrolyte, solvent, etc.). The TiO_2_NTAs synthesized by this Ti mesh can be used in many fields such as air purification, photoelectric catalysis, orthopedic targeted therapy, etc. And due to the hydrophilicity of the TiO_2_ film, they have a strong application prospect in dental implants [62].

## Figures and Tables

**Table 1:** The results of the EDS test.

Element		Ti	O
**content/wt%**	red area	59.481	40.519
	green area	95.228	4.772

## Data Availability

The original contributions presented in this study are included in the article. Further inquiries can be directed to the corresponding author.
